# Supplementary material for: Genome-Wide Identification and Analysis of Chitinase-Like Gene Family in Bemisia tabaci (Hemiptera: Aleyrodidae)
Source: Insects. 2021 Mar 17;12(3):254. doi: 10.3390/insects12030254 (PMC8002649; doi:10.3390/insects12030254)
Supplement: Supplementary file 1 [file insects-12-00254-s001.zip › Supplementary files/Table S1.docx]

| Gene | Description | Forward primer (5’-3’) | Reverse primer (5’-3’) |
| --- | --- | --- | --- |
| *BtCht8* | qRT-PCR | GCTACTCCCGCAAGCAGAAC | GCATTTTTACTCGGCGATGTC |
| *BtCht10* | qRT-PCR | GCTGCACCTCTGATGGTTACTATTC | ACTTTCTTTTCACTGTTCCACGCTA |
| *BtCht5* | qRT-PCR | CGGAACTTTGCGAAGACATC | TTGCCTTGGCTAAGCGTGA |
| *BtCht4* | qRT-PCR | AAACCTCGGTGGCGGAAT | AGCGGACAGAAAGTTCGTAAGA |
| *BtIDGF2* | qRT-PCR | CTCTCTCTGGCACGGAACAA | TGGAGATGGATAGAAGGTGGC |
| *BtCht6* | qRT-PCR | AACACCCGAACCAGTTACACCTT | GACTCTTGTGCTCGTGGATGG |
| *BtCht3* | qRT-PCR | ATACACAGGAAATGCAGCTAACG | GTATGCGGTGTAACCAACTTTTC |
| *BtIDGF3* | qRT-PCR | AAATGCCTCACGGAGACACC | CGACCGAATGTAGGGATGCT |
| *BtIDGF1* | qRT-PCR | GCCTCCGTCCGAGCATTAT | CCGCAGTCTTTTGGGTGTT |
| *BtCht2* | qRT-PCR | ATGACCAGGGGAAGGTGACA | GAAGCCGTAGATAAGGTGAGTGC |
| *BtCht9* | qRT-PCR | CACCTGGCAGCATTGAAAGA | GCGTAGATAGCATTGGTTAGGC |
| *BtCht7* | qRT-PCR | TGTAAAACGAGCGAACAATGC | TTTTTGTGGGAATCCTGGCT |
| *BtCht11* | qRT-PCR | TTGCTGCCCCTGCTTCTATC | CCCACTTGTGAACCGACCA |
| *BtENGase* | qRT-PCR | GCTGCTATGGTGGAGGAGAGTT | AGATGTTGAAAAAGGCAGGGAT |
| *BtRPL29* | qRT-PCR | TCGGAAAATTACCGTGAG | GAACTTGTGATCTACTCCTCTCGTG |
| *BtEF-1α* | qRT-PCR | TAGCCTTGTGCCAATTTCCG | CCTTCAGCATTACCGTCC |
| *BtCht8* | PCR | TCAAGGGAAGTGTCAAGTCA | AGATGCGCTAACTTCAATG |
| *BtCht10* | PCR | TTCCCGTTCCCTCCTCGTAA | CTGCCCAGACTACTTTCAGATTTT |
| *BtCht5* | PCR | GGCACAGTACAGTTCTTGACC | CTGTAAATCACCGTATGTGTGT |
| *BtCht4* | PCR | CTGGCTCGCTACTCGCTTCT | ATGAACAGAATGAAGGGAAGGC |
| *BtIDGF2* | PCR | CAGTCGAGCAATAAGTGAAA | GAGCCATGAATTGTAGTTCCTAAC |
| *BtCht6* | PCR | CTTTTCGGCAGTGTAGTGT | CAGTGTTCCGTGTCCGTA |
| *BtCht3* | PCR | AGGACTTACGGCTTCTTTAT | GTTGATACGGCGTTCTTC |
| *BtIDGF3* | PCR | GCGAGGCTCCGTGTAAA | CCAAGCATTCAGGGTTCT |
| *BtIDGF1* | PCR | TCAGACTTTCGCATCTTCAGC | CTCAAGTAACTGGTCGGATTCTC |
| *BtCht2* | PCR | TTTCGCCACATTCAGTTACTCTAC | AATCTGAGATGCGATGAGGACA |
| *BtCht9* | PCR | GAAAGTGTCTACAAGCAACCT | ATACGGCGTTCTCCATTA |
| *BtCht7* | PCR | CGACTCAATCTCGGCAGTT | CGTCTGTTGTGGAATATGGAG |
| *BtCht11* | PCR | TAATCTATTCAGCTTGTGCG | GGGGTGCATAACTTCGA |
| *BtENGase* | PCR | ATGAGCTGCAACAAGATAGT | TTACAAGCAAGATTCTCTACTG |
| *EGFP* | dsRNA synthesis | GGATCCTAATACGACTCACTATAGG  CAGTGCTTCAGCCGCTAC | GGATCCTAATACGACTCACTATAGG  GTTCACCTTGATGCCGTTC |
| *BtCht10* | dsRNA synthesis | GGATCCTAATACGACTCACTATAGG  CTCAATACACTGAAGCGCCA | GGATCCTAATACGACTCACTATAGG  TGGTACTGACCCCACAGACA |
| *BtCht5* | dsRNA synthesis | GGATCCTAATACGACTCACTATAGG  CAAACGTGGGAGGTACTCGT | GGATCCTAATACGACTCACTATAGG  TATGGGTGTCAGCAAATCCA |
| *BtCht7* | dsRNA synthesis | GGATCCTAATACGACTCACTATAGG  TTCACCCTAGTTGACCCGAC | GGATCCTAATACGACTCACTATAGG  AGCCTTGTCTGGGTGGTATG |

Table S1 Primers used in this study.
