# Supplementary material for: Genome-Wide Identification and Analysis of Chitinase-Like Gene Family in Bemisia tabaci (Hemiptera: Aleyrodidae)
Source: Insects. 2021 Mar 17;12(3):254. doi: 10.3390/insects12030254 (PMC8002649; doi:10.3390/insects12030254)
Supplement: Supplementary file 1 [file insects-12-00254-s001.zip › Supplementary files/Table S2.docx]

Table S2 PCR conditions for gene cloning.

| Temperature | Time |
| --- | --- |
| 95 ℃ | 10min |
| 95 ℃ | 30s  35 cycles |
| Tm | 30s  **35 cycles** |
| 72 ℃ | 1kb/min |
| 72 ℃ | 10min |
| 4 ℃ | Forever |
